# Signaling Mechanisms of Selective PPAR*γ* Modulators in Alzheimer's Disease

**DOI:** 10.1155/2018/2010675

**Published:** 2018-10-21

**Authors:** Manoj Govindarajulu, Priyanka D. Pinky, Jenna Bloemer, Nila Ghanei, Vishnu Suppiramaniam, Rajesh Amin

**Affiliations:** ^1^Department of Drug Discovery and Development, Harrison School of Pharmacy, Auburn University, Auburn, AL, USA; ^2^Center for Neuroscience, Auburn University, Auburn, AL, USA

## Abstract

Alzheimer's disease (AD) is a chronic neurodegenerative disease characterized by abnormal protein accumulation, synaptic dysfunction, and cognitive impairment. The continuous increase in the incidence of AD with the aged population and mortality rate indicates the urgent need for establishing novel molecular targets for therapeutic potential. Peroxisome proliferator-activated receptor gamma (PPAR*γ*) agonists such as rosiglitazone and pioglitazone reduce amyloid and tau pathologies, inhibit neuroinflammation, and improve memory impairments in several rodent models and in humans with mild-to-moderate AD. However, these agonists display poor blood brain barrier permeability resulting in inadequate bioavailability in the brain and thus requiring high dosing with chronic time frames. Furthermore, these dosing levels are associated with several adverse effects including increased incidence of weight gain, liver abnormalities, and heart failure. Therefore, there is a need for identifying novel compounds which target PPAR*γ* more selectively in the brain and could provide therapeutic benefits without a high incidence of adverse effects. This review focuses on how PPAR*γ* agonists influence various pathologies in AD with emphasis on development of novel selective PPAR*γ* modulators.

## 1. Introduction

Alzheimer's disease (AD) is the sixth leading cause of mortality in the United States. In 2018, an estimated 5.7 million Americans of all ages are living with Alzheimer's dementia and this is projected to increase to 14 million by 2050 [1]. However, there are limited options to prevent the progression of the disease. Moreover, the continuous increase in mortality rates due to AD reinforces the critical need for identifying novel molecular targets with therapeutic potential. For example, the failure of several recent potential therapies in clinical trials for improving cognitive deficits in AD by reducing amyloid beta (A*β*) levels, suggests a need to explore alternative approaches for AD treatment that are not focused upon altering A*β* levels.

Pathological correlations between type 2 diabetes mellitus (DM) and AD provide direct links for the development of cognitive deficits in both diseases and suggest potential application of antidiabetic drugs for AD [2, 3]. Type 2 DM is a disorder of altered glucose regulation and is associated with cognitive decline [4]. Although there are direct links between AD and DM in the manifestation of cognitive impairment, there is an understanding that impaired insulin signaling directly alters memory in AD. Insulin signaling in the brain has a significant role in modulating neuroendocrine and neurotrophic functions including synaptic plasticity [5, 6]. Therefore, extensive investigation of these correlations between the two diseases will potentiate the identification of novel therapeutic targets for the treatment of AD.

Peroxisome proliferator-activated receptors (PPARs), a subfamily of nuclear receptors, play a crucial role in regulating insulin sensitivity and may serve as potential therapeutic targets for AD. Recently, pharmacological activation by a class of PPAR subtype, PPAR*γ* agonists thiazolidinediones (TZDs), has been found to improve learning and memory in transgenic AD animal models [7, 8]. Furthermore, meta-analysis studies indicate that pioglitazone treatment may offer therapeutic benefits in patients with early or mild-to-moderate AD [9]. Further analysis of these studies showed a significant reduction in amyloid beta and tau pathology measured in cerebral blood flow samples from AD patients. The anti-inflammatory properties and improved glucose metabolism by TZDs have helped explain how it improves cognition in AD patients and transgenic animal models of DM and AD [10, 11]. However, the molecular signaling mechanisms mediated by central PPAR*γ* activation resulting in improved cognition in AD have not been extensively investigated. Furthermore, the use of these drugs for cognitive deficits in diabetes and AD is limited due to their poor bioavailability in the brain and off-target effects [12, 13]. Therefore, there is a critical need to develop PPAR*γ* targeted agents that display improved tolerability. To understand the significance of these chemical and pharmacological standpoints, the molecular structure and how PPAR*γ* modulates different cellular targets need to be more thoroughly evaluated.

Recently, the focus of PPAR*γ* has intensified, as new ligands and novel biological roles have emerged for the receptor activity, particularly for its therapeutic potential in neurodegenerative disorders, such as AD. The present review discusses the beneficial role of PPAR*γ* ligands on the pathologies of AD and the therapeutic potential of selective PPAR*γ* modulators as future therapy for AD.

## 2. Overview of PPARs

### 2.1. Isoforms and Expression

The PPARs are part of a subfamily of nuclear receptors that regulate several important cellular processes by activating or repressing transcription via their ligand binding domain (LBD) and DNA-binding domains (DBD) [14]. The initial PPAR (PPAR*α*) was cloned as a nuclear receptor from a mouse-liver genetic (cDNA) library that was activated by several endogenous and xenobiotic compounds known as peroxisome proliferators. PPARs are named for their property of increasing both the number and activity of liver peroxisomes after administering high dose of these substances for a chronic time frame in rodents. Additionally, marked liver abnormalities progressing to liver carcinomas were noted, indicating that these substances at high doses strongly induce peroxisome proliferation [15].

The PPARs are mainly divided into PPAR*α*, PPAR*β*/*δ*, and PPAR*γ*. All the PPARs consist of distinct functional domains including an N-terminal transactivation domain (AF1), a highly conserved DBD, and a C-terminal ligand binding domain (LBD) that contains a ligand transactivation function (AF2). Each subtype displays distinct effects on the body; for example, PPAR*α* regulates whole body energy homeostasis by reducing lipid levels, regulating glucose homeostasis, and reducing insulin resistance [16]. PPAR *β*/*δ* regulates lipid metabolism and myelination in the brain while PPAR*γ* regulates lipid and glucose homeostasis, mitochondrial biogenesis, and inflammation [17].

The PPAR*γ* receptor is unique in that despite being expressed from the same gene, it has different promoters and 5′-exons. Hence, it consists of three isoforms, namely, PPAR*γ*-1, PPAR*γ*-2, and PPAR*γ*-3. PPAR*γ*-1 and PPAR*γ*-3 are similar, while PPAR*γ*-2 differs in the ligand-independent region at the N-terminus. PPAR*γ*-2 has an extra 30 amino acid residues in the amino end, which provides a potent transcriptional activity compared to PPAR*γ*-1 [18]. PPAR*γ*-1 is expressed in almost all cells while PPAR*γ*-2 is restricted in the adipose tissue [19]. In the CNS (central nervous system), all the three subtypes of PPARs are expressed, with PPAR *β*/*δ* being the most abundant subtype [20, 21]. PPAR*α* is involved in acetylcholine metabolism, excitatory neurotransmission, and oxidative stress defense [22]. PPAR*β*/*δ* is ubiquitously expressed in all cell types including immature oligodendrocytes and promotes differentiation and myelination in the CNS [23, 24], while PPAR*γ* is expressed predominantly in microglia and astrocytes and regulates inflammation in the CNS [25].

### 2.2. PPAR Signaling

**Figure 1:**
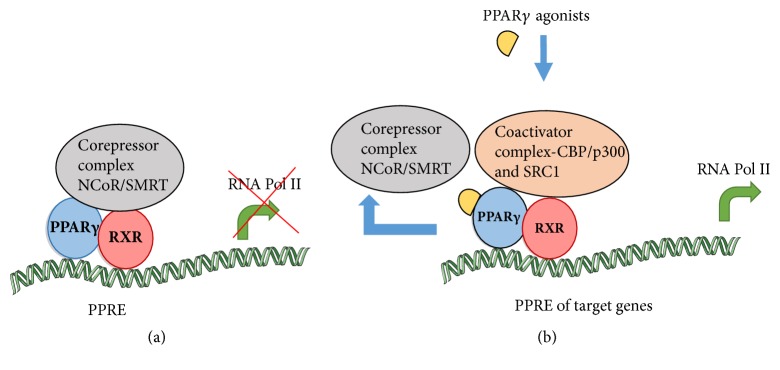
Mechanism of action of PPAR*γ* agonists. (a) During unstimulated conditions, the heterodimer is associated with corepressors which suppress gene transcription. (b) Binding of PPAR*γ* agonist induces release of corepressor complex, while binding to coactivator complex, thereby stimulating the response elements of target genes. This scaffold recruits histone acetyl transferase and RNA polymerase leading to transcription of target genes.

PPARs regulate the expression of various genes through a complex set of mechanisms. The homo PPAR forms a heterodimer with another class of nuclear receptors, retinoid X receptors (RXR). During unstimulated conditions, the heterodimer complex is associated with corepressors (NCoR and SMRT), which suppress gene transcription [26]. Ligand binding to the hydrophobic pocket of the PPAR receptor induces conformational changes in the LBD structure, thereby resulting in its activation. Changes in the conformation on ligand binding lead to release of corepressors NCoR/SMRT or Not1, which generally prevents gene transcription, respectively. Release of corepressor with full agonist results in the stabilization of the LBD, resulting in binding of coactivators CBP/P300, p160/SRC-1, and vitamin D receptor interacting protein (DRIP) or thyroid hormone receptor associated protein (TRAP) complexes resulting in the activation of the PPAR molecule. Once activated, the PPAR/retinoid X receptor heterodimer stimulates peroxisome proliferator response elements (PPRE) in the promoter region of target genes. This scaffold recruits histone acetyl transferases and the gene transcription machinery (RNA polymerase complex), which together initiate chromatin relaxation to permit transcription of target genes [27] as depicted in Figure 1. Coactivator PGC-1*α* gene expression is particularly important in mediating cognition and has shown protective effects against AD. In addition, it regulates mitochondrial biogenesis, oxidative metabolism, fatty acid oxidation, and gluconeogenesis via PPARs; these effects on mitochondria, in turn, can improve brain function [28].

**Figure 2:**
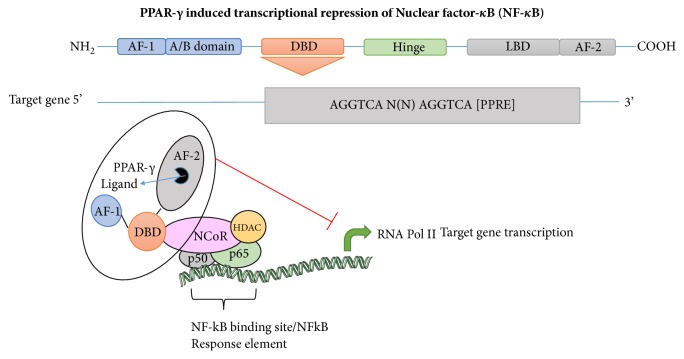
PPAR*γ* mediated transrepression of NF-*κ*B through SUMOylation modification. Binding of PPAR*γ* ligand to the AF-2 domain at Lys-365 position is important in regulation of inflammatory gene expression through transrepression. Additionally, recruitment of NCoR with inhibition of NCoR proteosomal degradation occurs.

Apart from the above-mentioned action of PPARs involving gene transcription in the nucleus, nongenomic actions associated with the cytoplasmic PPARs have been observed. Nongenomic regulation of PPAR*γ* is mediated by interaction with cytosolic second messengers, including kinases and phosphatases [29]. For instance, in response to mitogen stimulation, the MAP/ERK kinase, MAPK kinase (MEK)-1, binds directly to the AF2 domain of PPAR*γ*, leading to the sequestration of PPAR*γ* in the cytoplasm. Selective inhibition of this MEK1/PPAR interaction has been proposed to offer novel pharmacological treatments of various cancers, metabolic disorders, and inflammation [30]. Recent studies indicate that posttranslational control of PPARs occurs through phosphorylation, SUMOylation, ubiquitination, and nitration [31]. The phosphorylation of PPAR*γ* occurs at several sites via different kinases including MAPKs. An N-terminal serine phosphorylation (Ser82 in PPAR*γ*-1 and Ser112 in PPAR*γ*-2), mediated by MAPKs, reduces the transcriptional activity of PPAR*γ*-1 and PPAR*γ*-2 (in cells activated by serum) [32, 33]. Phosphorylation as a mechanism of action of PPAR*γ* agonists has been recently suggested linking its role to obesity, inflammation, and insulin resistance [34]. For instance, rosiglitazone was found to inhibit phosphorylation of PPAR*γ* at Ser273 by cyclin-dependent kinase 5 (CDK5) in adipose tissue. This leads to transcription of insulin-response genes (such as adiponectin and adipsin) thereby mediating antidiabetic effects. As CDK5 is activated by inflammatory mediators, targeting CDK5/PPAR*γ* regulation may offer new avenues in treating various disorders where inflammation is a key component. SUMOylation is a posttranslational modification that regulates the stability, nuclear/cytosolic ratio, and activity of several transcription factors. Of importance is the transcriptional repression of inflammatory genes such as inducible nitric oxide synthase (iNOS) and TNF-*α*, which are regulated by NF-kB pathway. Additionally, SUMOylation also induces recruitment of PPAR corepressors, such as NCoR as depicted in Figure 2.

The LBD of PPAR*γ* consisting of the transcriptional AF2 motif associated with helix 12 mediates most of the pharmacological actions of PPAR*γ* agonists [47]. The importance of AF2 motif in regulating PPAR*γ* targeted genes has been extensively studied, thereby allowing us to understand the mechanism of ligand-induced transcriptional activation by PPAR*γ* [48, 49]. AF2 helix is in an equilibrium state between closed (active) and open (inactive) conformations in the absence of the ligand [50]. However, binding to a full agonist leads to AF2 helix getting locked in closed (active) state, thereby allowing recruitment of coactivators for transcriptional activation [51]. Hence, developing novel PPAR*γ* ligands that stabilize the AF2 helix in distinct states between closed and open conformations would offer therapeutic advantage which is discussed in subsequent sections.

Several studies have reported that the locking of AF2 helix in its closed conformation is responsible for the antidiabetic effects as well as several side effects noted with PPAR*γ* agonists like TZDs [52–54]. Hence, developing novel PPAR*γ* ligands that stabilize the AF2 helix in distinct states between the closed and open conformations will selectively recruit coactivators for newer therapeutic benefits with reduced side effects [55–57].

Recently, several natural and synthetic PPAR*γ* agonists have been developed to treat various disorders, out of which the selective PPAR*γ* modulators (SPPAR*γ*Ms) have attracted considerable attention because of their ability to selectively target PPAR*γ* activity states, thereby offering therapeutic efficacy with minimal side effects [58–61]. Currently, no SPPAR*γ*Ms have been successfully used in clinical practice, and mechanistically it remains unclear how to achieve selective PPAR*γ* activation. The subsequent sections discuss the role of PPAR*γ* in modulating the pathologies of AD followed by SPPAR*γ*Ms under development for treating AD.

## 3. Overview of AD

**Figure 3:**
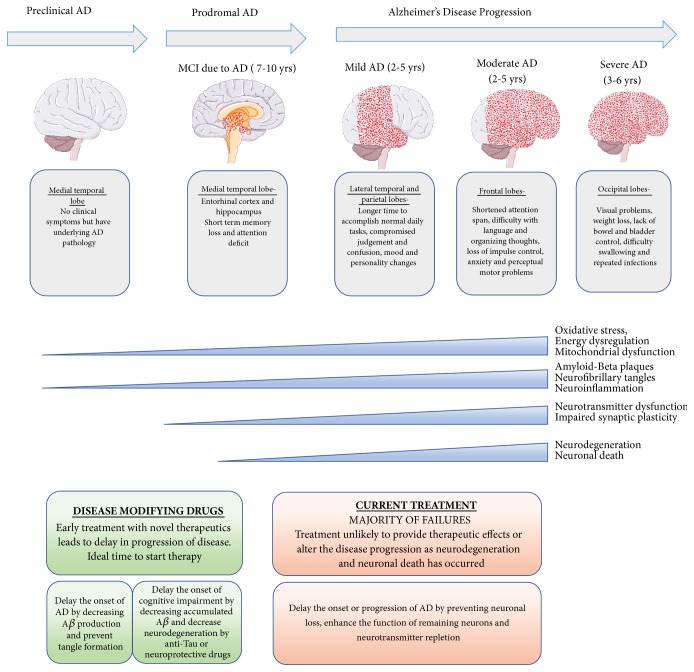
Description of progressive clinical stages and pathologies associated with Alzheimer's disease (AD) for relevance in developing therapeutic strategies for mitigating progression of AD.

Pathological changes related to AD occur many years before clinical symptoms are present. The traditional theory for the development of AD has been the amyloid beta cascade hypothesis, which postulates that pathogenic amyloid beta is the primary cause for development of AD and leads to the hyperphosphorylation of tau protein. However, it is becoming increasingly clear that a multitude of pathological mechanisms are likely at play to promote development of clinical AD [62]. Therefore, therapeutics with multiple mechanisms of action against AD pathology may be desirable in treatment of the disorder. Some early pathological processes which are increasingly recognized to contribute to AD include neuroinflammation [63], mitochondrial dysfunction [64], and dysregulated insulin signaling [65]. Amyloid beta accumulation occurs prior to clinical symptoms and persists throughout the course of the disease. Occurrence of memory dysfunction later in the disease is correlated with the severity of synaptic deficits and severity of tau pathology [66]. Therefore, an ideal AD drug may target multiple facets of the disease including inflammatory and metabolic components occurring early in disease, along with reducing pathogenic amyloid beta, hyperphosphorylated tau, and synaptic dysfunction later in the disease. In fact, PPAR*γ* signaling exerts several potential beneficial mechanisms in early, as well as in late, AD, as it reduces inflammation, improves metabolic processes, and may directly reduce levels of pathogenic amyloid beta and hyperphosphorylated tau. The hallmarks of AD relating to its pathologies are illustrated in Figure 3.

## 4. PPAR**γ** in Alzheimer's Disease

### 4.1. Genetic Alterations Relating PPAR*γ* to AD

Genome-wide association studies (GWAS) indicate a strong association between late onset Alzheimer's disease (LOAD) and over twenty genomic loci [67, 68]. One of the strongest genetic risk factors of LOAD is ApoE, and the presence of the ApoE4 allele is associated with an increased accumulation of A*β*. Several studies have shown increased ApoE-mRNA levels in LOAD brains and that cis-genetic variability contributes to differential ApoE gene expression [69–71]. Furthermore, chromosome 19q13.32, a gene rich region consisting of TOMM40, ApoE, and APOC1 genes, is implicated in several phenotypes including AD. This region exhibits a complex regulation and is enriched in potential PPAR*γ* binding sites. PPAR*γ* agonists decreased the levels of the TOMM40, ApoE, and APOC1-mRNAs, with the greatest effect on ApoE-mRNA through transcriptional regulation [72]. Furthermore, a study done by Barrera et al. investigated the effect of PPAR*γ* knockdown on expression of twenty-four late onset AD-associated genes and demonstrated that PPAR*γ* regulates the expression of seven LOAD-associated genes. Upregulation of six genes (ABCA7, ApoE, CASS4, CELF1, PTK2B, and ZCWPW1) and downregulation of one gene (*DSG2*) were noted and indicate that PPAR*γ* agonists may represent an attractive class of drugs for preventing or delaying the onset of late onset AD [73].

### 4.2. PPAR*γ* in Early Stages of AD

#### 4.2.1. PPAR*γ* and Amyloid Beta

AD is pathologically characterized by deposition of extracellular fibrillar amyloid derived from proteolytic cleavage of amyloid precursor protein (APP) and formation of senile plaques. An imbalance between A*β* production and its clearance leads to A*β* accumulation, which further leads to tau hyperphosphorylation and neurodegeneration [74]. PPAR*γ* agonists have also been observed to reduce A*β* levels either by reducing the A*β* production or enhancing its clearance. Several studies have demonstrated the role of PPAR*γ* agonists in decreasing A*β* accumulation. For instance, pioglitazone-treated APP transgenic mice showed reduced transcription and expression of *β*-secretase enzyme that processes APP to generate A*β* [75]. However, other studies have shown that APP processing and A*β* production are not affected by pioglitazone suggesting that decrease in A*β* levels by PPAR*γ* agonists may be due to A*β* clearance [76].

A*β* clearance in the brain is mediated by enzymatic and nonenzymatic pathways. Some of the key enzymes include insulin-degrading enzyme (IDE), neprilysin (NEP), and matrix metalloproteinase (MMP)-9. The nonenzymatic pathway includes (1) drainage through perivascular basement membranes, (2) phagocytosis by microglia or astrocytes, and (3) clearance mediated by receptors such as low-density lipoprotein receptor-related protein 1 (LRP1) and P-glycoprotein localized predominantly on the abluminal side of the cerebral endothelium [77, 78].

LRP1 is important in mediating endocytosis of various proteins such as ApoE and A*β*.

Low-density lipoprotein (LDL) receptor-related protein 1 (LRP1) is a transmembrane receptor involved in the endocytosis of more than 40 structurally different ligands, including ApoE, and A*β* peptide [79, 80]. Levels of LRP1 are found to be decreased in AD patients indicating its role in mediating A*β* metabolism. PPAR*γ* transcriptionally regulates LRP1 gene due to the presence of PPRE on the LRP1 promoter region [81]. A study by Rondon-Ortiz et al. reported that rosiglitazone transcriptionally activated LRP1 gene in a concentration dependent manner in HepG2 cells [82]. Similarly, rosiglitazone upregulated LRP1 expression at both the mRNA and protein levels, via PPAR*γ* activation [83].* In vivo* studies utilizing AD models need to be performed to validate the role of PPAR*γ* agonists and LRP1 in AD.

Apolipoprotein E (ApoE) is a lipoprotein expressed predominantly in the brain and is known to enhance A*β* degradation and phagocytosis in the microglia and astrocytes [74]. PPAR*γ* agonists (rosiglitazone and pioglitazone) have also been shown to induce A*β* degradation by increasing apolipoprotein E (ApoE) concentrations in the brain [76]. ApoE can increase the concentration of A*β* degrading enzyme neprilysin and insulin in astrocytes and microglia facilitating A*β* degradation [84]. In a recent study utilizing APP/Presenilin-1 mouse model [85], activation of PPAR*γ* enhances the microglial uptake of A*β* resulting in reduced cortical and hippocampal A*β* level. This effect is mediated by scavenger receptor CD36, which is a well-known player of phagocytosis. This finding can be further confirmed by observance of reduced microglial response to fibrillar A*β* response in CD36 null mice [86]. Additionally, pioglitazone treatment on APP/PS1 mice increased the levels of ATP-binding cassette transporter (ABCA1) and ApoE, thereby decreasing the levels of A*β* by 50%. In addition, the expression and processing levels of APP and of A*β*-degrading enzymes were not altered, suggesting that the changes seen in amyloid deposition were a result of A*β* catabolism [87]. Similar results were obtained with rosiglitazone treatment in a J20 mouse model [88] and with the APP/PS1 mice [89]. These studies suggest that TZDs enhance amyloid clearance in cell lines of microglia and astrocytes treated with A*β* and these effects are related to the activation of the ApoE pathway. Interestingly, rosiglitazone improved cognition in mild-to-moderate AD patients that did not carry ApoE4 allele. In contrast, no improvements were noted in cognitive test in ApoE4 positive patients, indicating that the amyloid clearance pathway dependent on TZDs also depends on the expression of functional ApoE4 [36]. The compelling results from animal models of Alzheimer's disease underline the beneficial effects of PPAR*γ* agonists on attenuating A*β* pathologies for future therapies.

#### 4.2.2. PPAR*γ* and Neuroinflammation

**Figure 4:**
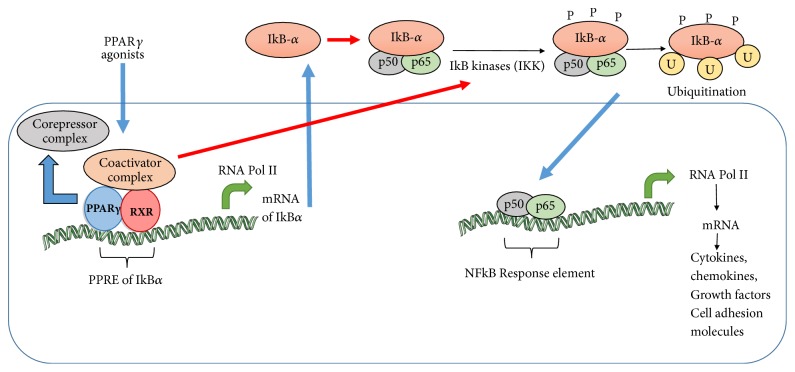
PPAR*γ* agonist mediated transrepression of NF-kB signaling. Activation of PPAR*γ* occurs upon binding of PPAR*γ* agonists through association of heterodimer with coactivator complex to form a transcriptional complex. This complex binds to the PPAR*γ* response element (PPRE) of IkB*α* gene, thereby inducing the expression of IkB*α*. An additional transrepressive mechanism involves inhibition of IKK*α*/*β*, an upstream kinase of IkB*α*. Consequently, degradation of IkB*α* and subsequent nuclear-translocation and activation of NF-kB (p50/p65) fail to occur, resulting in inhibition of inflammatory gene expression.

Several failures in AD clinical trials have encouraged researchers to look at treatment in the presymptomatic phase, where inflammation plays a vital role in the progression of neurodegeneration. One of the many potential beneficial effects of PPAR*γ* is its ability to downregulate inflammatory gene expression in immune cells [90, 91]. For example, PPAR*γ* activation has been shown to modulate the microglial response to amyloid deposition in such a way that it increases A*β* phagocytosis and decreases cytokine release [85]. The inflammatory hypothesis of AD involves activation of glial cells (microglia and astrocytes) by A*β*, which produces proinflammatory substances as a driving factor for neurodegeneration. Interestingly, a large meta-analysis reported that use of nonsteroidal anti-inflammatory drugs (NSAIDs), many of which have been shown to directly activate PPAR*γ*, is associated with a reduced risk of developing AD [92]. NSAID medications have provided key evidence that AD progression or initiation is related to neuroinflammation and that PPAR*γ* may mediate beneficial properties of NSAIDs in relation to AD. Commonly used NSAIDs including ibuprofen, indomethacin, and sulindac have been demonstrated to activate PPAR*γ* [93]. Under physiological conditions, the expression of PPAR*γ* in the brain is relatively less. However, its expression as measured by mRNA levels is elevated in AD patients, suggesting that PPAR may play a crucial role in modulating the pathology of AD [94]. Collectively, these findings led to the concept that PPAR*γ* could be an important target for mitigating brain inflammation in AD. More specifically, the activation of PPAR*γ* suppressed various transcription factors involved in inflammation such as nuclear factor-kB (NF-kB), Stat-1, and transcription factors activator protein-1 [95] which are important proinflammatory genes as illustrated in Figure 4.

Additionally, PPAR*γ* also downregulates cyclooxygenase-2 (COX-2), metalloproteinase-9 (MMP-9), inducible nitric oxide synthase (iNOS), proinflammatory cytokines, chemokines, and interleukins [96–98]. Thus, a reduction in PPAR*γ* activation may contribute to chronic inflammation, and pharmacological treatment with PPAR*γ* agonists may diminish expression of inflammatory genes. Several PPAR*γ* ligands, both natural (15d-PGJ2, docosahexaenoic acid) and synthetic (NSAIDs and TZDs), were shown to inhibit the expression of interleukin-6 (IL-6), TNF*α*, and cyclooxygenase-2 (COX-2) from monocytic and microglial cell cultures stimulated with A*β* [99]. Similarly, the anti-inflammatory effects of PPAR*γ* agonists like rosiglitazone and pioglitazone were noted in several AD mouse models [100, 101]. For example, the treatment with pioglitazone reduced astrocytes and microglial activation in the cortex and hippocampus of the A/T mouse that expresses high levels of A*β* and TGF-*β*1 [87]. At the same time, injection of rosiglitazone into the brain of Wistar rats, previously treated with A*β* oligomers, prevented the increase of inflammatory cytokines levels resulting in an improvement in cognitive decline and prevention of microglia activation [102]. Interestingly, similar effects were also observed in the AD transgenic mouse models J20 and APP/PS1 with oral administration of rosiglitazone [88]. Targeting microglia and astrocyte polarization may serve as therapeutic option. Cyclin-dependent kinase 5 (CDK5) is involved in activation of microglia and astrocytes and may serve as a potential therapeutic target for PPAR*γ* agonist therapy. This possibility is supported in a report where pioglitazone treatment, in conditional CDK5 knockout mice, displayed significant reduction in the activation of microglia and astrocytes and neuronal loss resulting in improved survival rates [103]. Mechanistically, CDK5 is a protein kinase, whose dysregulation contributes to synaptic loss and tau hyperphosphorylation in the AT8 epitope (present in the AD brain) after stimulation of A*β* fibrils [104, 105]. Together, these findings suggest that an anti-inflammatory property by TZDs involves regulating microglia and astrocytes inflammation and may involve CDK5.

Recently, PPAR*γ* has been implicated in macrophage polarization from M1, the classically activated phenotype, to M2, the alternatively activated phenotype, in several neurodegenerative diseases. M1 microglia have proinflammatory and neurotoxic properties through secretion of proinflammatory cytokines (interleukin IL-1*α*, IL-1*β*, TNF, and NO) [106, 107]. Alternatively, activated M2 microglia exhibit an anti-inflammatory phenotype and neurotrophic effect and degrade toxic aggregates due to anti-inflammatory interleukin production [106, 107]. The importance of PPAR*γ* in regulating the M1/M2 phenotypic switch has been confirmed by Amine Bouhlel et al., who demonstrated that activation of PPAR*γ* potentiates the polarization of circulating monocytes to macrophages of the M2 phenotype [108]. Subsequent studies reported that an active PPAR*γ* pathway is a prominent feature of alternatively activated (M2) macrophages and that M2-type responses are compromised in the absence of PPAR*γ* expression. PPAR*γ* expression is important for the full expression of certain genes characteristic of M2 macrophages, especially the gene encoding arginase-I, a direct PPAR target [109].

The small molecule SNU-BP was recently observed to inhibit inflammatory cytokine production and iNOS activity in LPS-stimulated microglia by PPAR*γ* activation [110]. In addition, SNU-BP also increased IL-4 and arginase-1 expression, which are considered as M2 microglial phenotype markers; thus SNU-BP further evaluation of* in vivo* testing will help decipher a novel mechanism for this compound for mitigating early AD. Conversely, 12-month-old APP/PS1 mice treated with pioglitazone showed a significant reduction of the immunofluorescence intensity of microglial activator marker M1 in the surrounding area of amyloid deposits and elevated expression of M2 markers. Reduction in the levels of GFAP-immuno-reactive astrocytes surrounding amyloid plaques and internalized A*β* peptides in astrocytes of pioglitazone-treated animals were noted. Together, these findings suggest that TZDs treatment not only reduced the inflammatory response by microglia and astrocytes but also facilitated the removal of A*β* deposits presumably through enhancing the phagocytic activity of these cells.

Interestingly, some of the anti-inflammatory effects of PPAR*γ* agonists appear to be independent of PPAR*γ* activity. For instance, the rank order potency of drugs that activate PPAR*γ* is often inconsistent with the anti-inflammatory efficacy, and expression of PPAR*γ* does not correlate with the observed anti-inflammatory effects. Moreover, part of the anti-inflammatory effects of TZDs is unaffected in PPAR*γ* knockout models, and secretion of both IL-6 and TNF-*α* is inhibited equally in both wild type and PPAR*γ* deficient macrophages [111]. Together, these data indicate that some anti-inflammatory effects of TZDs may be due to PPAR*γ* independent effects, thus suggesting that there exists a significant gap in understanding the mechanisms to explain how TZDs and PPARs confer their anti-inflammatory properties for AD and thus warrant further investigation.

#### 4.2.3. PPAR*γ* and Mitochondrial Function

Apoptosis, programmed cell death, is thought to be intimately involved in AD pathogenesis. A*β* localized to the mitochondrial membrane can initiate the intrinsic apoptotic pathway causing neuronal cell death [112]. Dysregulated metabolism results from altered mitochondrial trafficking and enhanced mitochondrial degradation, which can lead to alteration in tau and microtubular instability [113]. Increasing evidence suggests that PPAR ligands are involved in mitochondrial regulation in adipose tissue [114, 115] and other organs [116, 117] indicating a potential benefit against mitochondrial dysregulation in AD. PPAR-*γ* coactivator 1 alpha (PGC1-*α*) is highly expressed in the brain and acts as a critical transcriptional coactivator for mitochondrial biogenesis and cellular energy metabolism. Decreased hippocampal PGC1-*α* has been observed in postmortem analysis of AD patients [118]. Decreased PGC1-*α* expression has been reported to result in reduced mitochondrial density in various brain areas, including midbrain, cortex, and cerebellum accompanied with reduced ATP levels [119]. Both PPAR*α* and PPAR*γ* agonists prevent mitochondrial size reduction by enhancing PGC1-*α* expression in cultured hippocampal neurons [120]. In the APP23 mouse model of AD, PGC1-*α* gene delivery improves spatial and recognition memory along with reduction in A*β* level through a decrease in BACE1 activity [121]. However, excess PGC1-*α* can also exert deleterious effects via mitochondrial proliferation and produce toxicity in the heart [122], muscles [123], and brain, leading to cognitive impairment [124, 125]. Therefore, an ideal therapeutic approach would be to increase the PGC1-*α* level via an indirect mechanism, i.e., through PPAR ligands. Studies on N2A cells showed that treatment with rosiglitazone increased mitochondrial mass and function through the activation of PGC1-*α* mediated by the PKA/CREB/AMPK pathway [126]. In another study, chronic treatment with pioglitazone attenuated oxidative damage, restored mitochondrial respiratory activity, and enhanced mitochondrial biogenesis in Wistar rats injected with A*β* [127]. Taken together it can be stated that further research for novel therapeutic development of PPAR compounds will explore how these ligands will enhance mitochondrial function.

#### 4.2.4. PPAR*γ*, Insulin Signaling, and Brain Insulin Resistance

Insulin signaling is known to play a crucial role in the process of memory formation as insulin receptors are densely located in key areas of the brain, namely, olfactory bulb, hypothalamus, hippocampus, cerebral cortex, striatum, and cerebellum [128–131]. Insulin is essential to maintain normal neuronal homeostasis and survival, thus promoting learning and memory specially in the hippocampus [132]. For instance, brain specific insulin receptor knockout animal model showed increased tau hyperphosphorylation with altered Akt and GSK3*β* expression [133]. Additionally, diet induced insulin resistance in AD mice displayed increased A*β* peptide and advanced plaque formation [134]. These studies indicate that insulin resistance serves as an underlying mechanism for the development of A*β* production in the brain and associated sporadic memory impairment [135, 136].

Postmortem studies in AD patients have shown significantly reduced levels of insulin as well as insulin like growth factor (IGF-1 and IGF2) and insulin receptor substrate-1 (IRS-1) in the brain [137]. Abnormal insulin signaling leads to impaired neuronal oxidative metabolism [138]. The increase in oxidative stress and mitochondrial dysfunction can lead to aberrant posttranslational modification of APP and accumulation of A*β* in neurons [139]. Because of these shared features between AD and diabetes, AD has also been referred to as type III diabetes mellitus, leading to a growing interest in using insulin sensitizing agents as a potential therapy for AD [100, 140]. Rosiglitazone, a PPAR*γ* agonist, improved neuronal insulin resistance in high fat diet rat model by increasing the phosphorylation of AKT/PKB at Ser473. Additionally, high fat diet induced brain mitochondrial dysfunction and oxidative stress were attenuated by rosiglitazone [141].

The Wnt signaling pathway is important in mediating several functions in the central nervous system, including neuroprotection and synaptic plasticity, and deregulated Wnt signaling has been shown to be associated with AD [142]. Dysfunctional Wnt signaling is associated with A*β* deposition, tau hyperphosphorylation, and cognitive impairment as reviewed by Tapia-Rojas et al. [143]. In view of the energy dysregulation associated with AD, Wnt signaling has been shown to act as a central integrator of metabolic signals from peripheral organs to the brain, thereby promoting adequate glucose utilization in the neurons [144]. Furthermore, dysfunctional glucose utilization is also associated with several neurological disorders, indicating that Wnt signaling is important in AD pathogenesis [145, 146]. These findings suggest that pharmacological activation of Wnt pathway would be a feasible therapeutic approach for the treatment of AD.

### 4.3. PPAR*γ* in Later Stages of AD

#### 4.3.1. PPAR*γ* and Tau

Tau proteins are microtubule associated proteins that under normal physiological conditions interact with tubulin for microtubule assembly by mediating microtubule stabilization [147]. However, under pathological conditions such as AD, tau proteins undergo hyperphosphorylation resulting in neurotoxicity. Homeostasis maintained between kinase-mediated phosphorylation and protein phosphatases-mediated dephosphorylation is important in regulating the phosphorylation state of tau protein [148]. Various important kinases regulate tau phosphorylation, including cyclin-dependent kinases (CDK2 and CDK5), GSK-3*β*, mitogen-activated protein kinase (MAPK), extracellular signal-regulated protein kinase 1/2 (ERK1/2), c-Jun N-terminal kinase (JNK), Akt, protein kinase a (PKA), and calcium-calmodulin protein kinase 2 (CaMKII). Contrarily, PP1, PP2A, PP2B, and PP2C are important phosphatases that contribute to dephosphorylation of tau. Recent studies have shown that PPARs can also exert an effect on tau pathology.* In vitro* cellular studies using troglitazone and pioglitazone revealed reduced hyperphosphorylation of tau at Ser202, Ser396, and Ser404 in a tau transfected cell model involving GSK3*β* [149]. In the 3xTg-AD mouse model, treatment with pioglitazone resulted in significantly reduced tau phosphorylated-positive neurons in the hippocampus and improved cognitive deficits [150]. These studies also revealed that treatment with pioglitazone reduced tau phosphorylation at Ser202, Ser396, Ser404, Ser422, and Thr231 in cerebral cortex and CA1 area of hippocampus. Although these findings suggest that PPAR*γ* stimulation can reduce tau hyperphosphorylation, further mechanistic studies are needed to determine the signaling mechanism by which PPAR*γ* offers neuroprotection against tau hyperphosphorylation. Interestingly, the pan-PPAR agonist bezafibrate was shown to reduce tau phosphorylation in a P301 mice model by reducing iNOS and cyclooxygenase-2 in microglia [151]. Although role of PPAR*γ* in A*β* mediated pathogenesis is exhaustively studied, its role in tauopathy needs to be explored for potential mechanism for reducing the hyperphosphorylation in-depth. A great variation can be noticed in the existing literature which is likely due to variation in the animal model studied and the time point of study.

#### 4.3.2. PPAR*γ* and Synaptic Plasticity

Moderate to severe stages of AD are characterized by synaptic failure which leads to cognitive decline and memory dysfunction. Synaptic function is dependent on specialized structures on neuronal processes called dendritic spines, and the loss of dendritic spines directly correlates with the loss of synaptic function. One of the key mediators for increasing dendritic density and synaptic plasticity is neurotrophins, including brain-derived neurotrophic factor (BDNF) [152]. Several studies have reported beneficial effects of PPAR*γ* agonists in improving synaptic plasticity. Rosiglitazone has been shown to prevent dendritic spine loss and improve synaptic function in hippocampal neurons treated with A*β* oligomers [126]. Decreased expression of BDNF mRNA and protein levels were noted in hippocampi of db/db mice which were restored with rosiglitazone treatment. Furthermore, PPAR*γ* has been shown to transcriptionally regulate BDNF expression as demonstrated from promoter activity assays wherein ligand activation of PPAR*γ* induced the BDNF promoter in a log dose-dependent manner [153]. Similarly, A*β* injected rats treated with pioglitazone had reduced caspase-3 activation and increased BDNF levels, which was correlated with improved synaptic plasticity [127]. These observations suggest that PPAR*γ* agonists prevent the impairment of synaptic plasticity by increasing BDNF expression and dendrite spine density.

Numerous studies have reported that kinases, such as CDK5, are also vital in the regulation of synaptic plasticity [154]. For example, a recent study showed that pioglitazone, via a proteasome-dependent manner, decreased the expression levels of p35 resulting in reduced CDK5 activity in neurons [154]. Moreover, blockage of CDK5 by pioglitazone prevented long-term potentiation (LTP) defects at CA3-CA1 synapses in APP/PS1 mice [155]. Alternatively, rosiglitazone was reported to induce an increase in the expression of neurotrophic factor-*α*1 (NF-*α*1), a neuroprotective protein, which increases prosurvival protein BCL 2 expression and provides neuroprotection in hippocampus [156]. Neurotrophins, such as nerve growth factor (NGF), can also induce PPAR*γ* activation via the tyrosine kinase (TrkA) dependent signaling pathway and promote cell survival and differentiation [157]. PGC-1*α* gene therapy also increases NGF and exerts neuroprotective effects in AD mouse models [158]. These observations describe the significance of TZDs towards mitigating AD via improving mitochondrial function and synapse plasticity and reducing memory loss. Overall, PPAR*γ* activation can simultaneously promote mitochondrial functions, improve metabolic and energy regulation, modulate neuroinflammation, stimulate axonal growth and myelination, and clear toxic A*β* from the brain [159].

## 5. PPAR**γ** Agonists

### 5.1. Conventional PPAR*γ* Agonists: Drawbacks and Limitations

Rosiglitazone and pioglitazone have been recognized as potential treatments for the AD through their insulin sensitizing and anti-inflammatory effects [160]. Several clinical studies have tested the efficacy of TZDs treatments in AD [161]. However, some clinical trials utilizing pioglitazone have failed to show therapeutic benefit [162, 163]. A meta-analysis study showed insufficient evidence to support use of rosiglitazone in amnestic mild cognitive impairment and AD patients. Interestingly, pioglitazone showed efficacy especially in patients with comorbid diabetes mellitus [164]. However, another meta-analysis indicated that there is no statistically significant benefit with PPAR agonists in mild-to-moderate AD patients [165]. However, as previously stated, full agonists of PPAR*γ* mediated closed conformation of the AF2 helix is responsible for many TZDs side effects [52–54]. Some of the most common adverse effects noted with conventional PPAR agonists include edema and heart enlargement [35, 166]. Discovery and development of specific novel PPAR*γ* ligands with improved therapeutic profiles provide a molecular framework for future developments of pharmacological PPAR*γ* agonists with advantages over current TZDs drugs. Review of several clinical trials utilizing PPAR*γ* agonists in the treatment of AD has been summarized in Table 1, with references and highlights of the study.

### 5.2. New Direction for PPAR*γ* Agonists: Development of Selective PPAR*γ* Modulators (SPPAR*γ*Ms)

**Figure 5:**
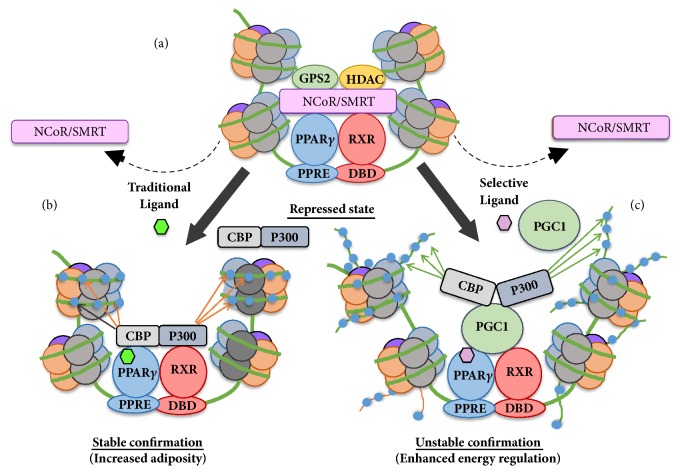
PPAR*γ* receptor (a) is in the repressed state due to the transcriptional cofactor inhibitors NCoR/SMRT binding with PPAR*γ* and preventing the transcription of target genes. When traditional (full agonist, (b)) or selective agonist (c) changes the confirmation of the PPAR*γ* receptor and corepressors NCoR/SMRT come off. Traditional agonists (full agonist) such as rosiglitazone or pioglitazone promote a stable confirmation of the PPAR*γ*-RXR confirmation with coactivators CBP/P300. However, selective agonists can induce an unstable confirmation of the PPAR*γ* complex and allow alternative interactions with nontraditional coactivators, potentially PGC-1*α*, thus inducing alternative gene expression.

Selective PPAR*γ* modulators (SPPAR*γ*Ms) have attracted considerable attention because of their ability to selectively target PPAR*γ* activity states. Several investigators have characterized and identified promising SPPAR*γ*Ms that serve as partial agonists for PPAR*γ* in cell based transcriptional activity and adipogenic assays [167, 168]. SPPAR*γ*Ms specifically bind to the LBD of PPAR*γ* via an activation function 2 motif (AF2). This offers greater flexibility in response to diverse ligands, resulting in different receptor conformations and coactivator and/or corepressor recruitment in different tissues [169]. Several SPPAR*γ*Ms in preclinical studies have demonstrated strong insulin sensitizing activity in diet induced obese C57/BL6 mice with attenuated adverse effects on adiposity, weight gain, and cardiac related complications compared to potent full PPAR*γ* agonists [170]. To further explain, the mechanism by which SPPAR*γ*Ms uniquely interact with the receptor results in diminished conformational stability of the receptor when compared to traditional TZDs. Co-crystallography studies of the PPAR*γ* LBD complexed with full PPAR*γ* agonist rosiglitazone demonstrated strong hydrogen bonding with the Tyr473 site in helix 12 of the human PPAR*γ* LBD. In contrast, rational drug design utilizing molecular modeling and crystallography structure analysis performed on the PPAR*γ* LBD with SPPAR*γ*Ms revealed that these compounds have the inability to form hydrogen bonding with Tyr473 due to the bonding distance with the carboxylic acid moiety [171, 172]. Findings from NMR studies have indicated that SPPAR*γ*Ms induce a less stable confirmation than full PPAR*γ* agonists (Figure 5) [173]. In addition, the Tyr473 site, within the helix 12 transcriptional activation function 2 domain, is involved in activation of the transcriptional coactivator binding pocket of the LBD [174]. Alteration of this site leads to the potential inability to directly stabilize this region and may serve as the physical basis for the differential receptor coactivator interaction, altered transcriptional activity, and reduced deleterious effects upon the heart and body associated with current full PPAR*γ* agonists. Traditional PPAR*γ* agonists, which display strong interaction with Tyr473, displace the inhibitory cofactors NCoR/SMRT and recruit P300/CBP, which confirms the most stable confirmation of the ligand binding pocket. These cofactors serve as histone acetylators and thus conform the receptor to the adipogenic gene in a stable confirmation. SPPAR*γ*Ms that lack interaction with Tyr473 induce less stable confirmation and thus allow predictions for alternative cofactor associations with the receptor including PGC-1*α*. The potential of SPPAR*γ*Ms in AD is just beginning and will likely lead to development of therapeutic targets for mitigating AD. Tyr473 in the AF2 region of the LBD has been shown to be a critical site of interaction between the full agonists and the PPAR*γ* receptor. Studies on mutant PPAR*γ*-LBD, where Tyr473 is replaced with alanine, revealed that interaction with Tyr473 is necessary for full agonist activity [175]. To this, various SPPAR*γ*Ms such as SPPAR*γ*M2, GW0072, INT131, and PA082 have been observed not to interact with Tyr473 residue. Recently, Bruning and coworkers demonstrated that several SPPAR*γ*Ms (BVT13, nTZDpa, MRL-20, MRL-24, SR145, and SR147) cause activation of PPAR*γ* by interaction and stabilization of the *β*-sheet and H3 rather than AF2 helix of the LBD, which acts as a novel coactivator interaction site [176]. It suggests that the structurally diverse SPPAR*γ*Ms, due to their distinct physical interaction with the receptor, uniquely bind to the receptor, resulting in diminished conformational stability compared with full agonists. Figure 5 illustrates the conformational changes in PPAR*γ*/RXR receptor induced by the binding of full agonists or SPPAR*γ*Ms, leading to coregulator heterodimer dissociation/recruitment, which forms the molecular basis for selective gene regulation that triggers specific metabolic effects. Studies with several SPPAR*γ*Ms have mainly focused on four families of coregulators: NCoR and silencing mediator for retinoid and thyroid hormone receptors (SMRT), the p300 and CREB binding protein (CBP) family, the PPAR*γ* coactivator 1 (PGC-1) family, and the p160 family, which are composed of three related coactivators (SRC1/NCoA1, GRIP1/TIF2/SRC2, and pCIP/RAC3/ACTR/AIB1/TRAM1/SRC3). Corepressors NCoR and Not1 and SMRT are known to downregulate full PPAR*γ* agonist-mediated transcriptional activity and inhibit adipogenesis [177]. Several SPPAR*γ*Ms that induce selective PPAR*γ* or lack displacement of corepressors have been observed to display partial or antagonistic effects on adipogenesis and yet maintained insulin sensitization. For example, telmisartan and halofenate, which act as a SPPAR*γ*Ms, induce reduced dissociation of corepressors and thus are partially adipogenic, while GW0072 (PPAR*γ* repressor) does not dissociate corepressors resulting in preventing adipogenesis. However, they all are effective insulin sensitizers. Alternatively, FK614 causes NCoR dissociation equal to that of rosiglitazone and is a full agonist in adipogenesis assay, but shows several characteristics of SPPAR*γ*M in vivo [173]. Since the corepressor dissociation studies with SPPAR*γ*Ms are limited and given that SPPAR*γ*Ms display diverse activity in adipogenesis assay, their effects on AD needs to be further explored with respect to corepressor interaction and its role in PPAR*γ*-mediated insulin sensitization. Coactivators CBP/p300, TIF2 and SRC-3 seem to favor fat accumulation [178], and therefore their recruitment may not be desirable, while the physiological role of other coactivators, such as SRC1 and PGC-1*α*, is more associated with energy regulation. Thus, the specific recruitment of TIF2 over SRC1 may be a reason for lipid accumulation observed following rosiglitazone treatment in diabetic patients [179]. Interestingly, MBX-102, a SPPAR*γ*M, promotes higher recruitment of CBP, TIF2, SRC1, and PGC-1*α* when compared to a full agonist [173]. Other SPPAR*γ*Ms including FMOC-leucine, PA-082, GW0072, and FK614 favor the recruitment of PGC-1*α* over that of SRC-1, TIF2, or p300 when compared with rosiglitazone [60, 180]. The role of PGC-1*α* is important for PPAR*γ* activity as it acts as a docking surface for integrating the actions of transcription factors and cofactors for regulation of mitochondrial biogenesis and oxidative capacity [181]. Also, it has been shown that rosiglitazone upregulates the constitutively low expression of PGC-1*α* in white adipose tissue [182]. In the aged brain, PGC-1*α* regulates the expression of sirtuin 3, which is a factor related to the aging process [183]. It has been observed that brains from patients with neurodegenerative diseases display low levels of PGC-1*α* which leads to mitochondrial dysfunction and oxidative stress [184]. PGC-1*α* regulates mitochondrial density in neurons and PGC-1*α*–knockout mice showed an increased sensitivity to the degeneration of dopaminergic and glutamatergic neurons in the brain [185]. Moreover, alternative studies have demonstrated that the reduction of mitochondrial gene expression in PGC-1*α*–knockout mice leads to neuronal dysfunction [186]. Given that PGC-1*α* plays a crucial role in neuronal function and regulates mitochondrial function, PGC-1*α* could ameliorate mitochondrial dysfunction and improve cognitive function in AD [118, 187]. Therefore, SPPAR*γ*Ms favoring the recruitment of PGC-1*α* may lead to the discovery of new drug therapy for AD. Some of the newer PPAR*γ* agonists in several disease models are listed in Table 2, with references and highlights of the study.

## 6. Conclusions and Future Directions

Following the utilization of PPAR*γ* agonists for type 2 diabetes mellitus in improving insulin sensitivity, the pleiotropic effects of PPAR*γ* in neurodegenerative diseases like AD have been increasingly investigated in recent years. Extensive research undertaken to improve the efficacy and/or safety of first-generation PPAR*γ* agonists (the TZDs) has led to a greater understanding of the complexity of PPAR regulation, specifically the importance of coactivator and corepressor proteins. Developing novel agonists that exploit cofactor biology to derive better agents and reduce the unwanted deleterious effects is currently in process. Recent efforts to demonstrate differential cofactor recruitment and to develop better preclinical efficacy/safety profiles of SPPAR*γ*Ms compared to conventional PPAR*γ* agonists are underway. Future directions in PPAR research are likely to focus on optimizing the PPAR subtype interaction profile, maximizing inhibition of PPAR*γ* phosphorylation, and screening against off-target activity.

## Figures and Tables

**Table 1:** Clinical trials of PPAR*γ* agonists.

Title	Treatment	# Of subjects	Methods	Inclusion criteria	Major outcome(s)	Results of major outcome(s)	Reference
***Efficacy of rosiglitazone in a genetically defined population with mild-to-moderate AD***	Rosi 2mg, Rosi 4mg, Rosi 8mg, or placebo daily for 24 weeks	511	RCT	Probable AD, MMSE score of 16-26	ADAS-Cog and CIBIC+	No significant difference; exploratory analyses suggested that ApoE4 non-carriers exhibited cognitive improvement with 8mg dose of Rosi	[35]

***Rosiglitazone monotherapy in mild-to-moderate AD: results from a randomized, double-blind, placebo-controlled phase III study.***	Rosi XR 2mg, Rosi XR 8 mg, donepezil 10mg, or placebo daiy for 24 weeks	693	RCT	Probable AD, MMSE score of 10-23	ADAS-Cog and CIBIC+	No significant difference for Rosi group vs. placebo; no interaction between treatment and ApoE status	[36]

***Rosiglitazone does not improve cognition or global function when used as adjunctive therapy to AChE inhibitors in mild-to-moderate AD: two phase 3 studies.***	Rosi XR 2mg, Rosi XR 8 mg, or placebo daily for 48 weeks in addition to an AChEI	2,822	RCT	Mild-moderate probable AD, MMSE score of 10-26	ADAS-Cog and CDR-SB	No significant difference; no interaction between treatment and ApoE status	[37]

***Efficacy of PPAR-*** **γ** *** agonist pioglitazone in mild AD***	Pio 15-30 mg daily or no treatment for 6 months	42	randomized, open-label	Mild AD, Clinical Dementia Rating score of 0.5 or 1	MMSE, ADAS-Cog	Significant improvement in MMSE and ADAS-cog from baseline in pio treated subjects	[38]

***Biomarker Qualification for Risk of MCI Due to AD and Safety and Efficacy Evaluation of Pioglitazone in Delaying Its Onset (TOMMORROW)***	Pio 0.8mg SR daily or placebo for up to 5 years	3,494	RCT	Cognitively normal patients	Time to diagnosis of MCI due to AD for subjects in the high-risk stratum	Trial discontinued due to inadequate treatment effect; full results not yet published	[39]

***Telmisartan vs. Perindopril in Hypertensive Mild-Moderate AD Patients (SARTAN-AD)***	Telmi 40mg, Telmi 80mg, perindopril 2mg, perindopril 4mg, or perindopril 8mg daily for 12 months	goal of 240	Randomized, open label	Probable AD or possible AD dementia due to concomitant cerebrovascular disease, MMSE 16-27	Ventricular enlargement, cognitive function based on ADAS-cog and cognitive battery	Trial is ongoing	[40]

***Effects of telmisartan on the level of A*** **β** ***1-42, interleukin-1*** **β** ***, TNF*** **α** *** and cognition in hypertensive patients with AD***	Telmi 40-80mg or amlodipine 5-10 mg daily for 6 months	48	Randomized	Probable AD and essential hypertension	ADAS-Cog and MMSE	Significant improvement in MMSE and ADAS-cog in Telmi treated subjects compared to amlodipine treated subjects	[41]

***Phase 2a Feasibility Study of T3D-959 in Subjects with Mild to Moderate AD***	T3D-959*∗* 3mg, 10mg, 30mg, or 90mg once daily for 2 weeks	36	Randomized, open-label	Mild-to-moderate AD, MMSE score of 14 -26	FDG-PET Imaging, ADAS-Cog	Trends towards improvement in ADAS-Cog	[42]

Clinical trials of PPAR*γ* agonists. AChEI: acetylcholinesterase inhibitor; AD: Alzheimer's disease; ADAS-Cog: Alzheimer's disease assessment scale cognitive subscale; ApoE: apolipoprotein E; CDR-SB: clinical dementia rating scale sum of boxes; CIBIC+: clinician's interview-based impression of change with caregiver input; FDG-PET: fluorodeoxyglucose-positron emission tomography; MMSE: mini mental status exam; PPAR-*γ*: peroxisome proliferator activated receptor gamma; Pio: pioglitazone; Rosi: rosiglitazone; Telmi: telmisartan; ∗T3D-959 is a dual PPAR*δ*/*γ* agonist.

**Table 2:** 

Ligand	Classification	Model	Amyloid	Other pathologies	references
**NP00111 and NP01138** **(Novel TZDs)**	PPAR*γ* agonist	cerebral cortex of embryonic day 18 rats	Decreased A*β*	Prevented activation of microglia and suppressed inflammatory markers. Restricted cortical or hippocampal neuronal cell death	[43]

**Pirinixic acid derivate MH84**	Dual gamma-secretase/PPAR*γ* modulator	Thy-1 A*β*PP_SL_ mice	reduced cerebral levels of A*β*40	NA	[44]

**INT131**	SPPARMs- non-thiazolidinedione compound	Rat primary hippocampal neurons	Improved neuronal survival against A*β*	increased dendritic branching, improved mitochondrial functions	[45]

**T3D-959**	Dual PPAR-*δ*/PPAR*γ* agonist	streptozotocin-induced AD mouse model	reduced A*β*	Reduced levels of oxidative stress, normalized expression of phospho-tau and choline acetyltransferase.	[42]
		Intracerebral streptozotocin (i.c. STZ) model	-NA-	Improved Brain Insulin/IGF Signaling and reduced neuroinflammation	[46]

PPAR*γ* agonists in various models of AD and its effects on various pathologies. Amyloid beta (A*β*), peroxisome proliferator-activated receptor gamma (PPAR-*γ*), thiazolidinediones (TZDs), streptozotocin (STZ).
